# Risk stratification by CMR in Chagas' disease

**DOI:** 10.1186/1532-429X-13-S1-P293

**Published:** 2011-02-02

**Authors:** Marly M Uellendahl

**Affiliations:** 1 Heart Institute (InCor-FMUSP) - São Paulo University, São Paulo, Brazil

## 

Chagas’ disease (CD) still remains an important health problem in Latin America. Cardiac involvement in CD is associated wiht a high mortality and morbidity and one of the major challenges is to identify high risk patients.Therefore a risk score to predict death in CD was recently developed and validated by Rassi and colleagues. Considering the hole of myocardial fibrosis(MF) on the pathogenesis of CD we believe that CMR(cardiovascular magnetic resonance) may become an important method to stratify patients with CD. We sought to evaluate morphological and functional characteristics, as well as the extent of MF in patients with CD by CMR and to search the prognostic value of MF detected by CMR, comparing these findings with the recently developed score of mortality (Rassi’s score).Thirty-nine seropositive patients for CD were studied , divided in two main groups: group I, without apparent cardiomyopathy consisted of 11 asymptomatic patients without signs of cardiac involvement, and group II, with apparent cardiomyopathy, consisted of 28 patients with known heart involvement. We excluded patients with previous myocardial infarction, clinical or laboratory evidence of ischemic heart disease, increased cholesterol levels, diabetes, heart valve disease, pacemakers, Implantable deffibrilators and vascular brain clips. All patients underwent CMR examination on 1,5T GE echo-speed, SYSTEM (Wakeusha, Wiscosin). Short and long-axis of the heart were obtained during breath-hold and triggered on eletrocardiogram pulse sequences. The first sequence was a gradient-echo (steady-sate free procession) to assess left ventricular (LV) and right ventricular (RV) morphology and function. The second sequence was an inversion-recovery prepared gradient-echo to obtain myocardial delayed enhancement (MDE) ,10 to 20 minutes after intravenous bolus of 0.2 mmol/Kg of gadolinium-based contrast. Regarding the morphological and functional analysis, significant differences were observed in both groups (p< 0.001). Our data supports that there is an inverse correlation between increasing Rassi’s score numbers and LV ejection fraction (r = -0.78 p < 0.001). Furthermore there was a strong correlation with the extent of MF and the Rassi’s score (r = 0.76, p < 0.001); and also with Rassi’s risk groups (low, intermediate and high risk) (5±7.7; 19.8±17.3; e 38.1±17.7%, respectively (p< 0,001, ANOVA). We concluded that CMR is an important technique in the evaluation of patients with CD, stressing morphological and functional differences in all clinical presentations. The strong correlation with the Rassi`s score and the extent of myocardial fibrosis detected by CMR, emphasizes its role in the prognostic stratification of patients with CD.

